# Lentivirus-mediated RNA interference of vascular endothelial growth factor in monkey eyes with iris neovascularization

**Published:** 2010-08-25

**Authors:** Meng-Ke Yuan, Yong Tao, Wen-Zhen Yu, Wang Kai, Yan-Rong Jiang

**Affiliations:** Department of Ophthalmology, People’s Hospital, Peking University, Beijing, China

## Abstract

**Purpose:**

To explore the in vivo anti-angiogenesis effects resulting from lentivirus-mediated RNAi of vascular endothelial growth factor (VEGF) in monkeys with iris neovascularization (INV).

**Methods:**

Five specific recombinant lentiviral vectors for RNA interference, targeting *Macaca mulatta* VEGFA, were designed and the one with best knock down efficacy (LV-GFP-VEGFi1) in H1299 cells and RF/6A cells was selected by real-time PCR for in vivo use. A laser-induced retinal vein occlusion model was established in one eye of seven cynomolgus monkeys. In monkeys number1, 3, and 5 (Group 1), the virus (1×10^8^ particles) was intravitreally injected into the preretinal space of the animal's eye immediately after laser coagulation; and in monkeys number 2, 4, and 6 (Group 2), the virus (1×10^8^ particles) was injected at 10 days after laser coagulation. In monkey number 7, a blank control injection was performed. In monkeys number 1 and 2, virus without RNAi sequence was used; in monkeys number 3 and 4, virus with nonspecific RNAi sequence was used; and in monkeys 5 and 6, LV-GFP-VEGFi1 was used.

**Results:**

In monkey number 5, at 23 days after laser treatment, no obvious INV was observed, while fluorescein angiography of the iris revealed high fluorescence at the margin of pupil and point posterior synechiae. At 50 days after laser treatment, only a slight ectropion uvea was found. However, in the other eyes, obvious INV or hyphema was observed. The densities of new iridic vessels all significantly varied: between monkey number 5 and number 3 (36.01±4.49/mm^2^ versus 48.68±9.30/mm^2^, p=0.025), between monkey number 3 and monkey number 7 (48.68±9.30/mm^2^ versus 74.38±9.23/mm^2^, p=0.002), and between monkey number 5 and number 7 (36.01±4.49/mm^2^ versus 74.38±9.23/mm^2^, p<0.001).

**Conclusions:**

Lentivirus-mediated RNAi of VEGF may be a new strategy to treat iris neovascularization, while further studies are needed to investigate the long-term effect.

## Introduction

Iris neovascularization (INV) and subsequent neovascular glaucoma (NVG) are serious complications for patients with retinal ischemia, which may attribute to central retinal vein occlusion, proliferative diabetic retinopathy or other ischemic retinal disorders [1].

Vascular endothelial growth factor (VEGF), an endothelial-cell-specific angiogenic factor whose production is increased by hypoxia, is considered to be the best studied key molecule in ocular neovascularization [2,3]. Elevated levels of VEGF have been identified in the aqueous humor of patients with rubeosis and NVG [4].

Panretinal photocoagulation (PRP) has became the most commonly-used therapy to obliterate newly formed vessels in varied ischemic retinal disease [1,5,6]. However, in patients with severe media opacity such as cataracts or vitreous hemorrhage, PRP can not be performed. In addition, common side effects of PRP include worsened visual field, decreased night vision, diminished color vision, and decreased contrast sensitivity. Therefore, it is necessary seek alternative or adjunctive therapeutic strategies in the treatment of ocular angiogenic diseases.

Recently, intraocular injections of VEGF antibody have shown encouraging outcomes for management of INV: intracameral or intravitreal injections of bevacizumab, a full-length antibody of VEGF, can lead to complete or partial reduction of INV in 92.8%—100% of subjects [7-9]. Nevertheless, due to the short half-life of bevacizumab, recurrence of INV can be observed as early as 4 weeks after injection [7], thus repeated multiple injections are necessary.

Therefore, one may postulate that the next improvement in treatment of INV would extend anti-VEGF therapy to allow stable and long-term suppression of the overactive VEGF pathway. RNA interference (RNAi) has emerged as a powerful tool to induce loss-of-function phenotypes by post-transcriptional silencing of gene expression [10]. In this study, in contrast to the relatively short-lived siRNA used in previous studies [11,12], we used a lentiviral vector expressing a small hairpin RNA (shRNA) in the monkey INV model system, which potentially allows longer term suppression, relative to ectopically administered RNA, siRNA, and oligonucleotides, of overexpressed VEGF due to the innate longevity of expression from integrated or episomally stable DNA vectors. Lentivirus vectors have been shown to express transgenes steadily and potently for months or years [13,14].

## Methods

### Lentivirus vectors for *VEGF* small hairpin RNA

**Figure 1:**
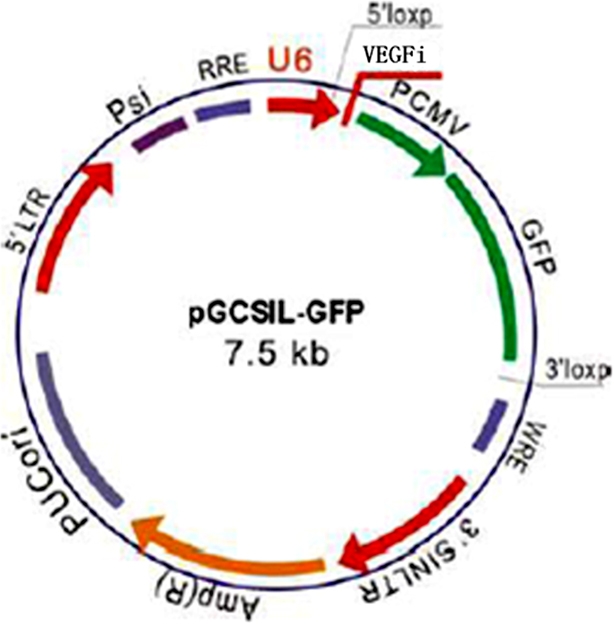
Construction of a lentivirus-mediated shRNA of *Macaca mulatta VEGFA*.

shRNA of *Macaca mulatta* VEGFA lentivirus gene transfer vector encoding green fluorescent protein (GFP) sequence was constructed by Genechem Co., Ltd, Shanghai, China (Figure 1). Five targeting sequences of the shRNA were designed as follows: 5′- AAT GCA GAC CAA AGA AAG ATA-3′ (VEGFi1), 5′-AGG GCA GAA TCA TCA CGA AGT-3′ (VEGFi2), 5′-CGA ACG TAC TTG CAG ATG TGA-3′ (VEGFi3), 5′-GAC GTG TAA ATG TTC CTG CAA-3′ (VEGFi4), and 5′-ATG CGG ATC AAA CCT CAC CAA-3′ (VEGFi5; GenBank XM_001089925). The lentivirus-GFP (LV-GFP) which included the *GFP* gene and did not include the VEGFA interference sequence served as negative control, and the target sequence, VEGFiCON (5′-TTC TCC GAA CGT GTC ACG T-3′), was designed with a randomly chosen nonsense sequence to serve as an additional control. The shRNA was confirmed by sequencing.

The recombinant lentivirus coding shRNA and targeting VEGFA (LV-GFP-VEGFi1, LV-GFP-VEGFi2, LV-GFP-VEGFi3, LV-GFP-VEGFi4, and LV-GFP-VEGFi5) and the negative control (LV-GFP-VEGFiCON and LV-GFP) were prepared and titered to 10^7^ TU/ml (where TU is transfection unit).

### Cell culture and infection

Established H1299 cells and a rhesus monkey choroid-retina endothelial cell line (RF/6A) were obtained from the ATCC (American Type Culture Collection, Rockville, MD). The H1299 cells and RF/6A cells were cultured in Dulbecco’s Modified Eagle Medium/F12 (Gibco, Grand Island, NY) with 10% fetal bovine serum. Cells were incubated at a constant 37 °C in a humidified incubator containing 5% CO_2_ −95% air. When the cells were about 50% confluent in complete DMEM medium, they were infected with the lentivirus constructs at different MOI (Multiplicity of Infection, or infectious unit ratio).

### Quantitative Real-Time PCR in vitro studies

To detect the efficiency of interference for VEGF, 1×10^6^ H1299 cells were transfected by LV-GFP-VEGFi(1–5), respectively, with 2×10^7^ (MOI=20, High MOI group) or 1×10^6^ (MOI=1, Low MOI group) particles. The LV-GFP-VEGFiCON and LV-GFP served as negative controls. The cells were harvested after 7 days for Quantitative Real-Time PCR of *VEGF*. The sequences of the *VEGF* primers were as follows: *VEGF* forward primer 5′-CAG ATG TGA CAA GCC GAG G-3′, *VEGF* reverse primer 5′-GCT GTC TAA TGC CCT GGA G-3′. Based on the result, the two LV-GFP-VEGFi with the highest knock-down efficiency were selected. In the second step, the two LV-GFP-VEGFi were used to transfect RF/6A cells in a similar way. Finally, the one sequence with the highest knock-down efficiency was selected for in vivo study.

### Experimental iris neovascularization

Seven cynomolgus monkeys (*Macaca fasicularis*) were used in this experiment in accordance with the Association for Research in Vision and Ophthalmology's Resolution on the Use of Animals in Research. Only the left eye was used. Using methods previously reported an induced ischemic branch retinal vein occlusion was performed to establish the iris neovascularization model [15]. The monkeys were anesthetized with an intramuscular injection of a mixture of 20 mg/kg bodyweight ketamine hydrochloride (Ketalar, Parke-Davis, Morris Plains, NJ), 1 mg/kg bodyweight of diazepam (Elkins-Sinn, Cherry Hill, NJ) and 0.125 mg/kg bodyweight of atropine sulfate (Dexter Corporation, Chagrin Falls, OH) for each examination and procedure. The eyes were gently protruded using an eye speculum and the pupils were dilated with 1% tropicamide [16]. Solid state laser light (532 nm, Novus spectra; Lumenis® Ltd, Santa Clara, CA) was used to occlude all branched retinal veins of the monkey eyes. The other laser parameters were: spot size 300 µm, power 300—360 mW, and exposure duration 200 ms.

### Monkey group information

After retinal vein occlusion, the monkeys were divided into two groups. In group 1 (monkeys number 1, 3, and 5), the virus (1×10^8^ particles per eye) was intravitreally injected into the preretinal space of the animal’s eye right after laser coagulation; and in group 2 (monkeys number 2, 4, and 6), the virus (1×10^8^ particles per eye) was injected at 10 days after laser coagulation (Table 1). In monkey number 7, the injection was performed but without actually injecting anything. The intravitreal injections were performed with a 26-gauge needle and the incision was made 2 mm behind the limbus. The needle was inserted tangentially toward the posterior pole of the eye, and about 100 µl of viral suspension was injected to the preretinal space. The injections were performed under a surgical microscope and the fundus could be observed during injection.

**Table 1:** The experimental treatment of the iris neovascularization model established in 7 monkeys.

**Monkey**	**Lentivirus type**	**Injection time after laser coagulation (days)**	**Group**
1	LV-GFP	right after laser	1
2	LV-GFP	10	2
3	LV-GFP-VEGFiCON	right after laser	1
4	LV-GFP-VEGFiCON	10	2
5	LV-GFP-VEGFi1	right after laser	1
6	LV-GFP-VEGFi1	10	2
7	sham control	fake injection	-

### Evaluation of iris neovascularization model

Iris neovascularization was evaluated by slit-lamp examination, iris photography, and fluorescein angiography of the iris (IFA). Angiography was performed with 10% sodium fluorescein (0.1 ml/kg bodyweight) via saphenous vein injection [16]. Fifty days after laser coagulation, animals were sacrificed by overdose of anesthesetic and the eyes were carefully enucleated and placed in 4% formalin(in 0.1 M PBS) overnight. The eyes were then grossly dissected and the nasal part of the globes was removed.

### Histopathological analysis of iris neovascularization

The globe was processed and embedded in paraffin. Serial sections were performed and stained with hematoxylin and eosin [17]. The section was immunohistochemically stained with antibody to factor VIII-related antigen/von Willebrand's factor (factor VIII) according to the methods of Noel Weidner [18]. Neogenic vessels are marked by factor VIII, but normal iridic vessels are not stained. Sections (5) were randomly selected from each group (eye) for factor VIII staining. Meanwhile, the calculation for positive INV on each section was conducted under the microscope; the iris section area was calculated with software Scion Image. The INV density on each iris section could be derived based on the two above parameters per mm^2^.

### Statistical analysis

Statistical analysis was performed using a commercially available statistical software package (SPSS for Windows, version 16.0; SPSS, Chicago, IL). The independent *t*-test was used to compare the density of iridic new vessels between groups. The data were distributed normally and are presented as the mean±SD. All p-values were two-tailed and were considered statistically significant when the values were less than 0.05.

## Results

### Comparison of interference efficacy between different designs of lentivirus-GFP-shRNA and control

**Figure 2:**
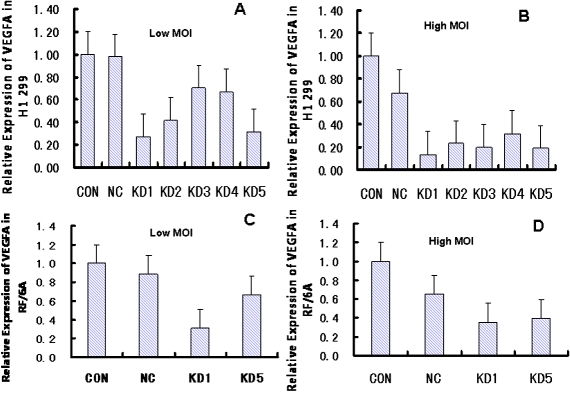
Gene silencing by lentiviral delivery of *VEGFA* siRNA. In the study with H1299 cells (**A** and **B**), expression of *VEGFA* was lower after blocking by siRNA sequences KD1 and KD5 than the other three sequences, under both the low MOI and high MOI. In the study with RF/6A cells (**C** and **D**), expression of *VEGFA* was lower after blocking by siRNA sequence KD1 than KD5, under both low MOI and high MOI. In the High MOI group, MOI=20, which means that 1×10^6^ cells were transfected by 2×10^7^ particles of virus. In the Low MOI group, MOI=1, which means that 1×10^6^ cells were transfected by 1×10^6^ particles of virus (CON: blank control; NC: negative control; KD1–5: knock down 1–5; MOI: Multiplicity of Infection).

All five LV-GFP-VEGFi were tested in vitro. First, the Real-Time RT–PCR in H1299 cells demonstrated that LV-GFP-VEGFi1 and LV-GFP-VEGFi5 are the most efficient shRNA. According to the ratio of shRNA to control, the VEGF levels were all decreased by more than 70% either in the high MOI or the low MOI group (Figure 2A,B and Table 2). Second, we tested LV-GFP-VEGFi1 and LV-GFP-VEGFi5 in the RF/6A cells, and the results obviously indicated that LV-GFP-VEGFi1 could inhibit expression of *VEGF*, and was superior to LV-GFP-VEGFi5 (Figure 2C,D and Table 2): in the high-MOI group (Figure 2D), the LV-GFP-VEGFi1 and LV-GFP-VEGFi5 knock-down efficiencies were 64.2% and 60.8%, respectively; in the low-MOI group (Figure C), the knock-down efficiencies were 69.1% and 33.7%, respectively. Third, we packaged the screened shRNA and selected LV-GFP-VEGFi1 to co-transfect 293T cells with pHelper 1.0 and Helper 2.0; the high titer Lentivirus were harvested and purified for in vivo experiments [19].

**Table 2:** Interference efficacy of lentivirus-GFP-shRNA in cells.

		**CON**	**NC**	**KD1**	**KD2**	**KD3**	**KD4**	**KD5**
H1 299	MOI=1	1.00±0.06	0.98±0.10	0.27±0.08	0.42±0.17	0.71±0.08	0.67±0.18	0.31±0.09
	MOI=20	1.00±0.13	0.68±0.16	0.14±0.05	0.23±0.11	0.20±0.08	0.32±0.10	0.19±0.04
RF/6A	MOI=1	1.00±0.18	0.88±0.22	0.31±0.13	–	–	–	0.66±0.19
	MOI=20	1.00±0.12	0.66±0.25	0.36±0.11	–	–	–	0.40±0.03

The values of this table represent the ratio of VEGF expression level in each group to control.

### Establishment of the INV model

**Figure 3:**
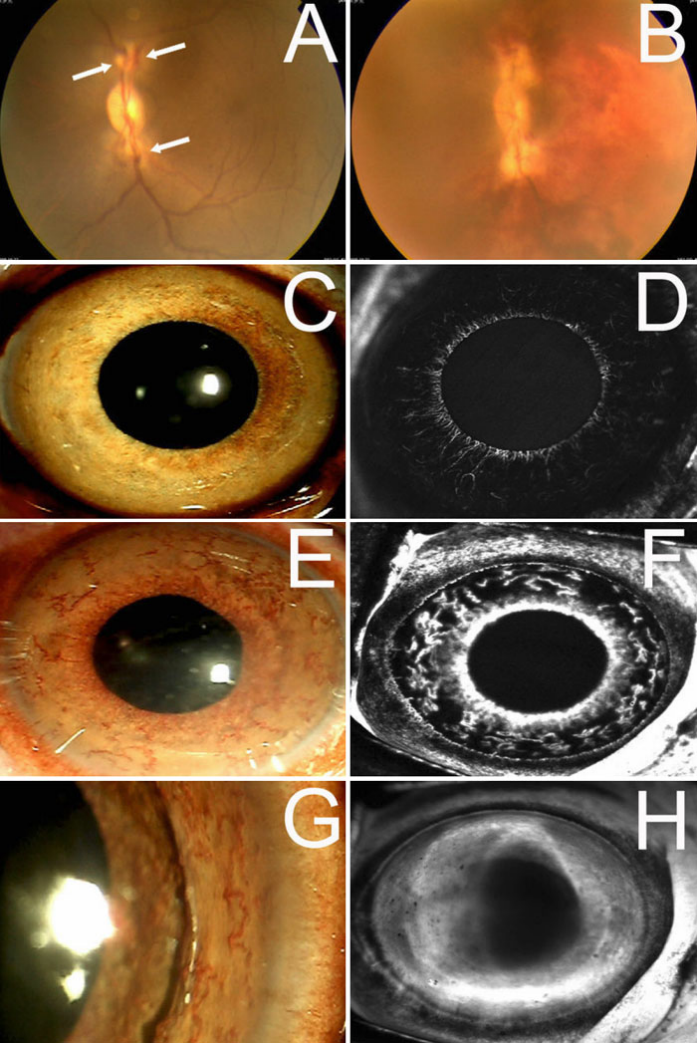
Color photograph and fluorescein angiography of the iris of monkey number 7 which underwent fake intravitreal injection. Laser irradiation was performed to occlude all major branched retinal veins of the monkey’s eyes (**A**). Obvious venous dilation and corresponding retinal hemorrhage can be observed from 2 days after laser coagulation (**B**); at 7 days after laser coagulation, mild iris neovascularization can be observed at the margin of pupil (**C**) with slight leakage of fluorescein (**D**); and at 14 days after laser coagulation, severe and tortuous iris neovascularization can be observed across the whole iris (**E**) with strong leakage of fluorescein (**F**) which lasted to 23 days after laser coagulation (**G** and **H**).

Laser irradiation was successfully performed to occlude all major branched retinal veins of the monkeys’ eyes (Figure 3A). In monkey number 7, which served as sham control, obvious venous dilation and corresponding retinal hemorrhage can be observed from 2 days after laser coagulation (Figure 3B); at 7 days after laser coagulation, mild iris neovascularization can be observed at the margin of the pupil with slight leakage of fluorescein (Figure 3C,D); and at 14 days after laser coagulation, severe and tortuous iris neovascularization can be observed at the whole iris with strong leakage of fluorescein (Figure 3E,F), which lasted to 1 month after laser coagulation (Figure 3G,H).

### Effect of *VEGF*-RNAi

Severe bacterial endophthalmitis occurred in monkeys number 1 and number 2 at 3 days after intravitreal injection of LV-GFP. Therefore, the data of monkey number 1 and number 2 were discarded.

**Figure 4:**
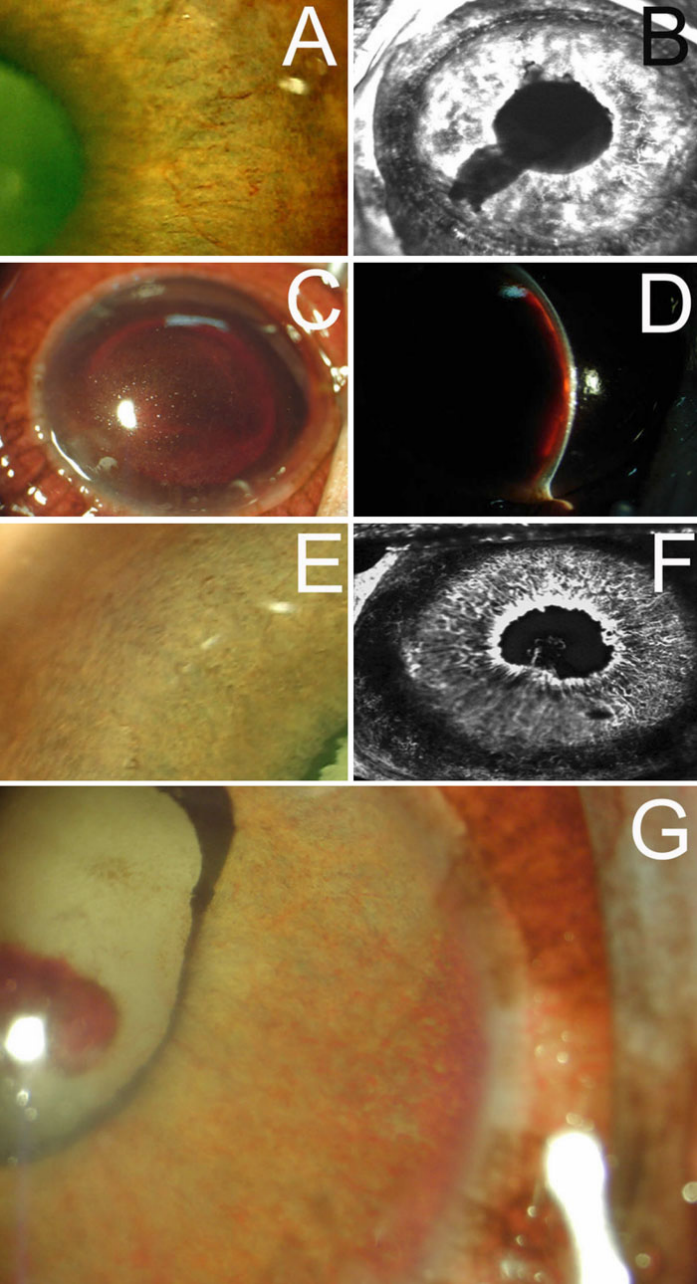
Color photograph and fluorescein angiography of irises of monkeys at 23 days after laser coagulation. In monkey number 3, moderate iris neovascularization (**A**) can be observed through the whole iris with obvious leakage of fluorescein (**B**). In monkey number 4, severe hyphema prevented the observation of iris (**C** and **D**). In monkey number 5, no obvious iris neovascularization (**E**) can be observed, while IFA revealed high fluorescence at the margin of pupil and point posterior synechiae (**F**). In monkey number 6, thin iris neovascularization can be observed through the whole iris together with an irregular and fixed pupil. Ectropion uveae and hemorrhages adhering to the anterior surface of lens were present (**G**).

**Figure 5:**
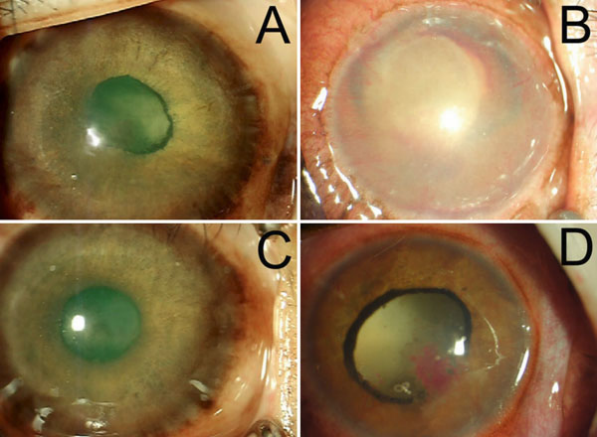
Color photograph iris of monkeys at 50 days after laser coagulation. In monkey number 3, un-regressed new blood vessels still can be observed throughout the whole iris (**A**). In monkey number 4, secondary glaucoma occurred (severe corneal epithelium edema was present; **B**). In monkey number 5, slight ectropion uvea was found (**C**). In monkey number 6, new thin iris vessels can be observed through the whole iris together with an irregular and fixed pupil. Ectropion uveae were apparently observed (**D**).

In monkey number 3, at 23 days after laser coagulation, moderate iris neovascularization was observed throughout the whole iris with obvious leakage of fluorescein (Figure 4A,B). At 50 days, un-regressed new blood vessels could still be observed throughout the whole iris with obvious leakage of fluorescein at IFA, but the leakage was less severe than that observed at day 23. Meanwhile, severe ectropion uvea could be observed (Figure 5A).

In monkey number 4, after laser coagulation, severe hyphema prevented the observation of the iris (Figure 4C,D). At 50 days, secondary glaucoma occurred (severe corneal epithelium edema was present; Figure 5B).

In monkey number 5, at 23 days after laser coagulation, no obvious iris neovascularization was observed under slit-lamp examination (Figure 4E), while IFA revealed high fluorescence at the margin of pupil and point posterior synechiae (Figure 4F). At 50 days, IFA revealed a less severe leakage of fluorescein. A slight ectropion uvea was found (Figure 5C).

In monkey number 6, after laser coagulation, new thin iris vessels were observed throughout the whole iris together with an irregular and fixed pupil. Ectropion uveae and hemorrhages adhering to the anterior surface of lens were present (Figure 4G). At 50 days, the findings were similar that hemorrhage, new thin iris vessels, an irregular and fixed pupil, and ectropion uveae were observed (Figure 5D).

For a better illustration of in vivo experiments, we summarized the results in different groups and stages, and tabulated these data in Table 3.

**Table 3:** Imaging observation results of anterior segment of monkeys.

**Group**	**No.**	**Days**	**INV**	**Leakage of fluorescein**	**Posterior synechia of pupil**	**Ectropion uvea**	**NVG**	**Hyphema**
1	3	23	+	+++	+	–	–	–
		50	+	+	+	–	–	–
	5	23	–	++	+	–	–	–
		50	–	+	–	–	–	–
2	4	23	*	+++	*	*	–	+++
		50	++	++	++	+++	++	–
	6	23	++	++	++	+	–	+
		50	+	+	++	+	–	–
CON	7	23	+++	+++	–	–	–	–
		50	++	++	+	++	+	–

In the table, INV indicates iris neovascularization and NVG indicates neovascular glaucoma. The asterisk indicates that severe hyphema prevented the observation of the iris.

### Histopathological examination of iris

**Figure 6:**
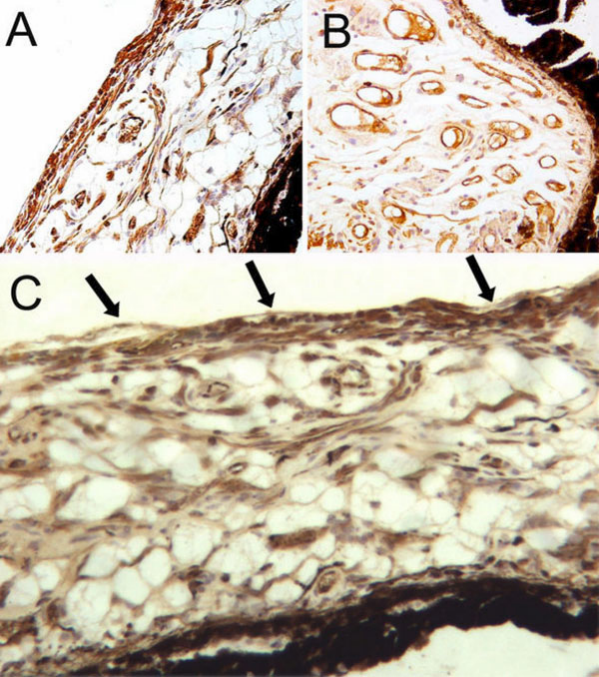
Immunohistochemistry of VIII factor for number counting of new vessels of iris. The density of iridis new vessels in the monkey number 5 (**A**) was 36.01±4.49/mm^2^ and 74.38±9.23/mm^2^ in monkey number 7 (**B**). In monkey number 3, a remarkable fibrosis membrane at the anterior surface of iris was observed (arrows, **C**). The original magnification was: 50× for **A** and **B**, and 100× for **C**.

Table 4 showed the density of iris new vessels counted microscopically. The density of iridic new vessels were lowest in monkey number 5 which underwent intravitreal injection of LV-GFP-VEGFi1 immediately after laser coagulation of the retinal veins (36.01±4.49/mm^2^) and highest in monkey number 4 which received an intravitreal injection of LV-GFP-VEGFiCON at 10 days after laser coagulation of retinal veins (77.12±10.79/mm^2^). In monkeys number 3, number 6, and number 7, the density of iridic new vessels were 48.68±9.30/mm^2^, 68.14±9.87/mm^2^, and 74.38±9.23/mm^2^, respectively. Figure 6 A,B showed the new vessels of iris in monkey number 5 and number 7. A remarkable fibrosis membrane at the anterior surface of iris in monkey number 7 and number 3 (Figure 6C), but it was not observed in other monkeys.

**Table 4:** Density of new iris vessels in monkey eyes (n=1).

**Monkey**	**No. 3**	**No. 4**	**No. 5**	**No. 6**	**No. 7**
RNAi injected	VEGFiCON	VEGFiCON	VEGFi1	VEGFi1	sham injection
Group	Group 1	Group 2	Group 1	Group 2	
Density of INV	48.68±9.30/mm^2^	77.12±10.8/mm^2^	36.01±4.49/mm^2^	68.14±9.87/mm^2^	74.38±9.23/mm^2^
Comparison of density of INV					
Between monkeys					
No. 3	–	–	p=0.025	–	p=0.002
No. 4	–	–	–	p>0.05	p>0.05
No. 5	p=0.025	–	–	–	p<0.001
No. 6	–	p>0.05	–	–	p>0.05
No. 7	p=0.002	p>0.05	p<0.001	p>0.05	–

The following differences in new iridic vessel density were significant: between monkey number 5 (group 1) and monkey number 3 (group 1; p=0.025), between monkey number 3 (group 1) and monkey number 7 (blank; p=0.002), and between monkey number 5 (group 1) and monkey number 7 (blank; p<0.001).

There were no significant difference observed between monkey number 4 (group 2) and monkey number 6 (group 2), between monkey number 4 (group 2) and monkey number 7 (blank), and between monkey number 6 (group 1) and monkey number 7 (blank) in density of new iridic vessels (p>0.05).

## Discussion

Results showed that iris neovascularization morphologically regressed in the monkey which underwent intravitreal injection of lentiviral vector expressing anti-*VEGF* shRNA immediately after laser coagulation of branched retinal veins to establish INV model. However, severe iris neovascularization or ectropion uvea were observed in the monkey which underwent intravitreal injection of lentiviral vector expressing anti-*VEGF* shRNA at 10 days after laser coagulation of branched retinal veins. Accordingly, the density of new iridic vessels was significantly lower in the eye with immediate intravitreal injection of shRNA-LentiVirus than the control eye with fake intravitreal injection, and lower than the monkey eye with intravitreal injection of shRNA-LentiVirus at 10 days after establishment of INV.

The present results were consistent with a recently reported study that used small interference RNA targeting *VEGF* to inhibit corneal neovascularization in the rat model [20]. In this study, corneal neovascularization was induced by cauterization with sodium hydroxide in rat corneas. The *VEGF*-siRNA-transfected corneal epithelium cells were transplanted to CNV lesions. Immediately after transplantation, the *VEGF*-siRNA combined with lipofectamine were directly injected into the anterior chamber to transfect the rat cornea. The authors found that the levels of expression of both *VEGF* mRNA and protein in the *VEGF*-siRNA transfected corneal epithelial cells and keratocytes were significantly lower than in controls. The new-vessels-occupied surface areas of the *VEGF*-siRNA-transfected-corneal epithelium transplantation group were also significantly less than the control group.

A similarly positive outcome was observed by Murata et al. [21]. They found that exposure to diced siRNAs significantly reduced *VEGF* mRNA expression in ARPE-19 cells with minimal toxicity. In suture-induced corneal angiogenesis models, diced siRNAs minimized the severity of angiogenesis.

In addition, using RNA interference to block the expression of *VEGF* was reportedly effective for choroidal neovascularization [11], and diseases other than ocular angiogenic diseases, such as colorectal cancer [22,23] and breast carcinoma [24].

Since the efficacy and mode of delivery of siRNA, a therapeutic tool, vary considerably [25], one should take the method of delivery into account. Chemically or enzymatically synthesized siRNA is costly and has been shown to have a relatively short half-life, with only transient inhibition of target genes [26]. Repeated administration of siRNA can compensate for the reagent's short half-life with the drawback that the risk of complications of intraocular injections increase. It has been reported that lentiviruses can efficiently transduce cells [27] and lentiviral delivery of siRNA can allow for efficient and stable gene silencing [28], therefore, we tested the efficacy of RNAi for *VEGF* by lentiviral delivery in our study.

Interestingly, obvious regression of iris neovascularization was only observed in the monkeys which underwent intraocular injection of lentiviral delivery of shRNA for *VEGF* immediately after laser coagulation of branched retinal veins. Severe iris neovascularization or ectropion uvea were still present in the monkey which underwent the same injection at 10 days after laser coagulation of branched retinal veins. One may infer that although virus-mediated RNAi for *VEGF* can achieve a long-term and stable gene silencing, it may be effective only if used at the early stages of retinal ischemia. The mechanism of action needs more investigation to reveal why this time difference occurs.

The limitations of this study should be mentioned. First, the number of monkeys used in this study was small. Therefore, data for incidence of complications with minor risks of intraocular use of lentiviral delivery of shRNA for *VEGF* were not obtainable, and the present results can only be regarded as preliminary. Second, the follow-up time was relatively short. The long-term effect of this kind of strategy should be investigated in future studies.

In summary, we demonstrated an encouraging and positive preliminary outcome of early injection of lentivirus-mediated siRNA for *VEGF* in treatment of iris neovascularization in the monkey model. Further studies should be performed to investigate the long-term effect of this strategy.
